# Post-Stress Fructose and Glucose Ingestion Exhibit Dissociable Behavioral and Physiological Effects

**DOI:** 10.3390/nu11020361

**Published:** 2019-02-09

**Authors:** Michael A. Conoscenti, Nicole M. Williams, Lorraine P. Turcotte, Thomas R. Minor, Michael S. Fanselow

**Affiliations:** 1 Department of Psychology, University of California, Los Angeles, CA 90095, USA; nwilliams12@g.ucla.edu (N.M.W.); mfanselow@gmail.com (M.S.F.); 2 Department of Biological Sciences, University of Southern California, Los Angeles, CA 90007, USA; turcotte@usc.edu; 3 Department of Psychiatry & Biobehavioral Sciences, University of California, Los Angeles, CA 90095, USA; 4 Staglin Center for Brain & Behavioral Health, University of California, Los Angeles, CA 90095, USA

**Keywords:** glucose, fructose, liver glycogen, CBG, cortisol, learned helplessness, PTSD, rat

## Abstract

An acute traumatic event can lead to lifelong changes in stress susceptibility and result in psychiatric disease such as Post-Traumatic Stress Disorder (PTSD). We have previously shown that access to a concentrated glucose solution for 24 h beginning immediately after trauma decreased stress-related pathology in the learned helplessness model of PTSD and comorbid major depression. The current study sought to investigate the peripheral physiological effects of post-stress glucose consumption. We exposed 128 male Sprague-Dawley rats to inescapable and unpredictable 1-milliamp electric tail shocks or simple restraint in the learned helplessness procedure. Rats in each stress condition had access to a 40% glucose solution, 40% fructose solution, or water. Blood and liver tissue were extracted and processed for assay. We assessed corticosterone, corticosteroid-binding globulin (CBG), glucose, and liver glycogen concentrations at various time points following stress. We found that rats given access to glucose following exposure to traumatic shock showed a transient rise in blood glucose and an increase in liver glycogen repletion compared to those that received water or fructose following exposure to electric shock. We also found that animals given glucose following shock exhibited reduced free corticosterone and increased CBG compared to their water-drinking counterparts. However, this difference was not apparent when glucose was compared to fructose. These data suggest that post-stress glucose prophylaxis is likely not working via modulation of the HPA axis, but rather may provide its benefit by mitigating the metabolic challenges of trauma exposure.

## 1. Introduction

Exposure to traumatic stress results in a number of physiological and psychological changes in both human and non-human species [1,2]. These changes are often deleterious in nature and can endure throughout a lifetime. As such, there is an urgent need for practical interventions aimed at treating or preventing the damaging effects of traumatic stress.

The learned helplessness procedure is a classic model used to analyze the behavioral symptoms of Post-Traumatic Stress Disorder (PTSD) and comorbid depression related to an acute, traumatic stressor in rats [3,4,5,6,7]. The procedure consists of two phases, which are an acute-traumatic shock phase and a testing phase that occurs 24 h later. In the initial phase, rats are either exposed to 100 inescapable and unpredictable shocks over an extended period, or restrained in plexiglass tubes for that same interval. All rats are then tested 24 h later for escape-performance in a shuttle box. Rats that receive inescapable shock show a profound, exaggerated fear response and shuttle-escape deficits during testing [8,9,10]. This transition to an unresponsive, depression-like state is referred to as conservation-withdrawal [11].

A number of findings suggest that metabolic homeostasis is challenged by exposure to uncontrollable, traumatic stress [8,9,10,12,13]. Minor and Saade (1997) hypothesized that simply treating rats with glucose following traumatic stress would restore energy homeostasis and eliminate the helplessness effect [14]. They found that shocked rats given 18-h access to a 40% (wt/vol) aqueous glucose solution immediately following traumatic shock stress no longer exhibited exaggerated fear responding and escape latency deficits in the shuttle-box. However, the mechanism by which glucose exerts its prophylactic effects has yet to be investigated.

Several studies have indicated that corticosteroids (CORT, cortisol in humans and corticosterone in rodents) are necessary to develop learned helplessness [15,16]. Uncontrollable stress causes elevation in CORT, which creates abnormalities in the hypothalamic-pituitary-adrenocortical axis [17]. Metyrapone blocks CORT synthesis and upregulates CORT catabolism. Injection of metyrapone before inescapable shock prevents learned helplessness [18,19], which illustrates that the stress-induced rise in CORT is necessary for the development of the learned helplessness phenotype. 

The actions of corticosteroids are not only modulated by production and release of this hormone via the HPA axis. In fact, 95% of cortisol is bound under resting conditions [20,21]. Approximately 80% of CORT is bound to the high-affinity, low capacity corticosteroid-binding globulin (CBG), 15% bound to the low-affinity, high capacity albumin, with the remaining 5% consisting of its free (or “freed”) form. Qian et al. (2011) showed that CBG regulated levels of free CORT in rats during a stressor [22]. CBG binds corticosterone to produce a functionally inactive form [23]. CBG is also released from the liver when blood glucose levels have risen [23]. Therefore, we hypothesize that post-trauma glucose ingestion may upregulate CBG protein synthesis, which allows for the increased binding of circulating glucocorticoids. This downregulates free CORT.

An alternative explanation is that the prophylactic effects of glucose are independent of glucocorticoid action and merely lie in its ability to prevent the negative metabolic sequelae of trauma. Rats receive inescapable, unpredictable shock transition from an initial anxious reaction to an inactive, depression-like state when exposed to test stimuli [8,9,24]. This state serves as an adaptive mechanism for husbanding limited resources and facilitating the recovery of metabolic homeostasis [11] and is likely mediated by brain adenosine signaling [6,8,9,25]. Given that energy expenditure dramatically increases on a total-brain scale when the animal is in a state of fear [26,27,28] and glucose transport is impaired during a stress response [12], it is possible that glucose is simply mitigating the negative metabolic impacts of stress.

This study used the learned helplessness procedure to examine the physiological impacts of post-stress glucose consumption. This study aimed to examine the impact of glucose for reducing the circulating levels of free CORT, increasing CBG, and increasing liver glycogen following stress pre-treatment and time of testing.

## 2. Materials and Methods

### 2.1. Subjects

**Figure 1:**
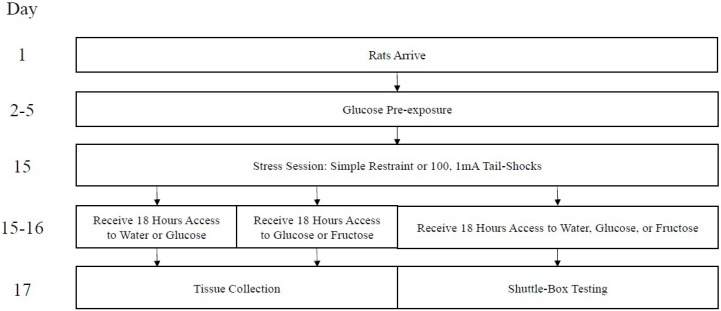
Timeline of events. Day 1 coincides with postnatal day (PND 50), approximately.

One hundred twenty-eight Sprague-Dawley albino male rats (290–320 g) from Envigo (Placentia, CA, USA) were housed in individual cages in a room maintained on a 12:12-h light/dark cycle (6:00–17:59 lights on, 18:00–5:59 lights off). Animals were housed in the room for approximately two weeks prior to testing. During this time, all animals had free access to water and food. All experimentation took place during the early light cycle (7:00–10:00, approximately). A timeline of all procedures is presented in Figure 1. The protocols in this paper received pre-approval by the UCLA Institutional Care and Use Committee.

### 2.2. Apparatus

Rats were housed in metal hanging cages. Each cage was equipped with a standard glass (250 mL) water bottle with a rubber stopper and metal spout.

Rats were restrained in Plexiglass clear restraining tubes during stress pre-treatment, as previously described [24]. Unscrambled electric shock was administered via electrodes attached to a rat’s extended tail. Each restraining tube was housed during the session in an illuminated, sound-attenuating chamber. Testing occurred in a shuttle box, as previously described [24]. A metal barrier divided the shuttle box into equal chambers. The chamber contained a center-pivoting grid floor that delivered scrambled shock. 

### 2.3. Procedure

Rats were assigned randomly to groups of eight rats each. Every group was pre-exposed to a glucose cocktail and a fructose cocktail over four consecutive days [14]. The cocktails consisted of 40% glucose or fructose and 5% sucrose dissolved in tap water (weight/volume).

Rats were exposed to either inescapable shock or simple restraint, which was followed by free access to glucose, water, or fructose. Twenty-four hours following stress pre-treatment, one cohort of rats was sacrificed via rapid decapitation. Trunk blood and liver samples were collected for later analysis. It should be noted that, after evidence of a dissociable behavioral effect between post-stress glucose and fructose, a fructose group was later added to this analysis and all samples were compared to a glucose group using a new cohort of rats. Another group of rats was exposed to the same stress pre-treatment and fluid access as above, but underwent serial blood draw before and after stress pre-treatment. These same animals also underwent testing 24 h later.

We exposed half of the groups (S: shocked) to 100, 1.0 mA variable-duration (mean = 8.0 s, range: 3 to 15 s), and inescapable tail shocks on a variable-time 60-s schedule (range: 20 to 150 s) in restraining tubes during a 110-min stress pre-treatment session. The other groups (R: restrained) were restrained in tubes for the same period and received no shock. Groups received free access to water (W: Groups SW and RW), glucose (G: Groups SG and RG), or fructose (F: Groups SF and RF) for 18 h beginning immediately following the pre-treatment stress session. We recorded total fluid consumption during this interval. All rats had free access to water over the final 6 h.

Testing or tissue collection occurred 24 h after the pre-treatment stress session in all groups. Testing began with five FR-1 trials on a 60-s fixed-time schedule. These trials required a rat to cross from one chamber to the other to terminate foot shock. During the inter-trial interval, freezing was assessed using a six-second time-sampling procedure. Freezing is defined as total immobility of the animal [29]. Twenty-five FR-2 trials on a 6-second variable time schedule (range: 20–230 s) followed three minutes after FR-1 trial completion. These trials required a rat to cross from one chamber to the other and back to terminate foot shock. Shock was terminated on a given trial after 40 s if the animal did not meet the response contingency. Latency to terminate shock was recorded for each FR-2 trial. The intensity of shock was set at 0.6 mA.

### 2.4. Plasma Sample Analyses

Blood was collected from the tail prior to the acute stress session and 0, 3, and 6 h following the acute stress session in one group of animals. At the time of typical testing (24 h after stress pretreatment), another group of rats was sacrificed using a small rat guillotine. Blood was collected from the trunk of the rat and the right lateral lobe of the liver was extracted. 

Assay of CBG, free corticosterone, and total corticosterone plasma concentrations were determined by using a commercially-available ELISA kit (Cat# E-EL-R1112, Elabscience, Bathesda, MD, USA; ADI-900-097, Enzo Life Sciences, Farmingdale, NY, USA). The assays were performed according to the manufacturer’s instructions. Liver tissue was pulverized using an electric pestle [30]. To prepare liver tissue for the glycogen assay, we followed procedures for hydrolysis [31], standard preparation [32], and analysis of tissue [33]. The concentration of CBG is presented as ng/mL of plasma, free and total corticosterone as ug/dL, and glycogen as ug/g of tissue, which accounts for the dilution factor.

### 2.5. Statistical Analysis

Software package SPSS (SAS Institute, Inc., Version 16.0, Cary, NC, USA) was used for statistical analyses. A multivariate analysis of variance (MANOVA) with stress type and fluid type as the between-subjects factors was conducted for free and total corticosterone plasma concentrations. A mixed-design ANOVA with stress type and fluid type as the between subjects factors was conducted for post-stress glucose consumption and CBG plasma concentrations. *A priori* planned comparisons were also made to determine whether inescapable tail-shock would reduce liver glycogen concentrations, and if post-stress glucose would replenish these depleted stores. Following significant interactions, Neuman-Keuls post-hoc analysis are reported. Statistical significance was noted when *p* values were less than 0.05. Data is presented as group means with error bars denoting group mean +/− SEM. No statistical outliers were removed from the data. Animals were excluded solely based on equipment malfunction.

## 3. Results

### 3.1. Effects of Post-Stress Glucose on Peripheral Physiology at the Time of the Test

Baseline glucose consumption for individual rats ranged between 21 and 45 mL. Mean intake was similar among groups and across pre-exposure days. A mixed-design analysis of variance (ANOVA: Group × Pre-exposure Day) yielded no statistically significant main effects or interactions, F(3,69) = 0.798, *p* = 0.499. Post-stress fluid consumption ranged between 15 and 48 mL. A single-factor ANOVA showed no statistically significant effect of group, F(3,69) = 1.398, *p* = 0.251.

**Figure 2:**
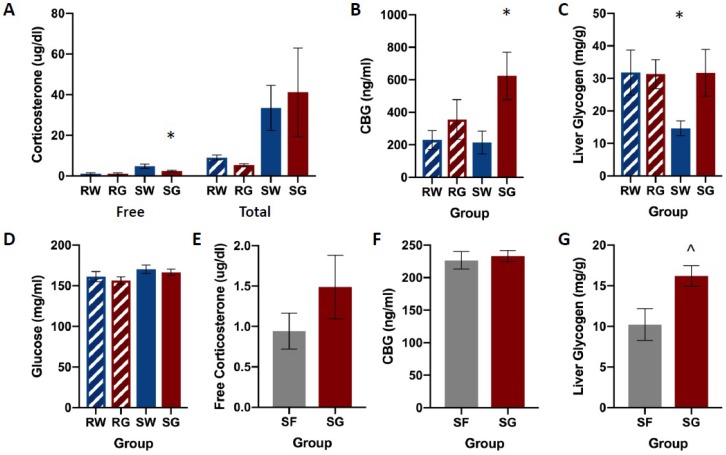
Corticosterone (panels **A**,**E**), CBG (panels **B**,**F**), liver glycogen (panels **C**,**G**), and glucose (panel **D**) concentrations among groups, following FR-1 shuttle-escape testing. Animals received either inescapable and unpredictable shock (S) or simple restraint (R). Following the stress session, animals were given 18-h free access to a 40% glucose cocktail (G), 40% fructose cocktail (F), or water (W). In shocked rats, glucose reduced free CORT, increased plasma CBG, and increased liver glycogen compared to water controls. However, CBG and corticosterone concentrations did not differ between shocked rats that received glucose or fructose. Liver glycogen concentrations were higher in shocked rats that received glucose compared to their fructose-drinking counterparts. Error bars denote mean ± SEM. * *p* < 0.05 (comparison: SG, SW), ^ *p* < 0.05 (comparison: SG, SF).

Figure 2 shows free and total corticosterone, CBG, and liver glycogen concentrations among groups. Shock groups showed much higher concentrations of both free and total corticosterone compared to their restraint counterparts. Restraint groups showed no differences in free or total corticosterone levels regardless of the type of solution they consumed (Figure 2A). Shocked rats that received glucose following the stress session (SG) showed decreased concentrations of free corticosterone compared to shocked rats that received only water. Shocked rats showed no differences in total corticosterone levels regardless of the solution consumed. The water groups (RW & SW) showed lower concentrations of CBG compared SG (Figure 2B). RG showed modest, but not significant elevations of CBG compared to both water groups. The group that received the traumatic shock condition followed by *ad libum* access to water (SW) showed much lower liver glycogen concentrations compared to all other groups (RW, RG, SG, Figure 2C). No other groups appear to differ in liver glycogen concentrations. Groups did not differ in blood glucose concentrations (Figure 2D), F(3,26) = 1.584, *p* = 0.217.

A multivariate ANOVA on corticosterone concentrations yielded a significant main effect of Group on Free CORT, F(3,28) = 20.039, *p* < 0.001, as well as a significant main effect of the Group on Total CORT, F(3,28) = 5.032, *p* < 0.001. Neuman-Keuls post-hoc comparisons (α = 0.05) on group means indicated a relationship among groups for Free CORT, such that: RW = RG < SG < SW. Neuman-Keuls post-hoc comparisons (α = 0.05) on group means indicated a relationship among groups for Total CORT, such that: RW = RG < SW = SG.

A one-way ANOVA on CBG concentrations yielded a significant main effect of Group, F(3,28) = 3.384, *p* = 0.034. Neuman-Keuls post-hoc comparisons (α = 0.05) on means indicated a relationship among groups such that: RW = RG = SW < SG.

*A priori* planned comparisons using two-tailed t-tests were conducted to compare restraint and shock conditions (RW, SW), and glucose and water groups within the shock condition (SW & SG). Unpaired, two-tailed t-tests showed a significant difference in liver glycogen between RW and SW groups, t(14) = 2.31, *p* = 0.036, and between SW and SG groups, t(14) = 2.52, *p* = 0.025. 

Also pictured in Figure 2 are identical measures assayed in a new cohort of rats that received either glucose or fructose following shock. Baseline glucose and fructose consumption for individual rats ranged between 20 and 31 mL. Mean intake was similar among groups and across pre-exposure days. A single-factor analysis of variance (ANOVA: Group) yielded no statistically significant main effects for glucose, F(1,13) = 0.394, *p* = 0.541, or fructose, F(1,10) = 3.954, *p* = 0.075. Post-stress fluid consumption ranged between 20 and 47 mL. A single-factor ANOVA showed no statistically significant effect in the group, F(1,14) = 3.384, *p* = 0.087. 

No group differences were observed for free corticosterone or CBG plasma concentrations (Figure 2E,F). However, the SG group showed a significantly higher concentration of glycogen in the liver compared to SF (Figure 2G).

Single-factor ANOVA yielded no statistically significant effects of the group on free corticosterone, F(1,14) = 2.292, *p* = 0.152, or CBG, F(1,14) = 0.174, *p* = 0.683. A single-factor ANOVA analysis yielded a significant effect of the Group on liver glycogen concentrations, F(1,12) = 5.917, *p* = 0.032.

### 3.2. Effects of Post-Stress Glucose on Peripheral Physiology Following Stress Pre-Treatment

Baseline glucose and fructose consumption for individual rats ranged between 16 and 37 mL. Mean intake was similar among groups and across pre-exposure days. A mixed-design analysis of variance (ANOVA: Stressor × Fluid Type × Pre-exposure Day) yielded no statistically significant main effects or interactions for glucose, F(2,50) = 0.516, *p* = 0.600, or fructose F(2,42) = 0.928, *p* = 0.403. Post-stress fluid consumption ranged between 1 and 4 mL per hour. A mixed-design ANOVA (Stressor × Fluid Type × Time Bin) yielded statistically significant interactions of Time Bin by Stressor, F(2,92) = 6.689, *p* = 0.002, and Time Bin by Fluid Type, F(4,92) = 10.313, *p* < 0.001. Newman–Keuls post-hoc comparisons (α = 0.05) indicated the following order of relationship among group means: W = F < G.

**Figure 3:**
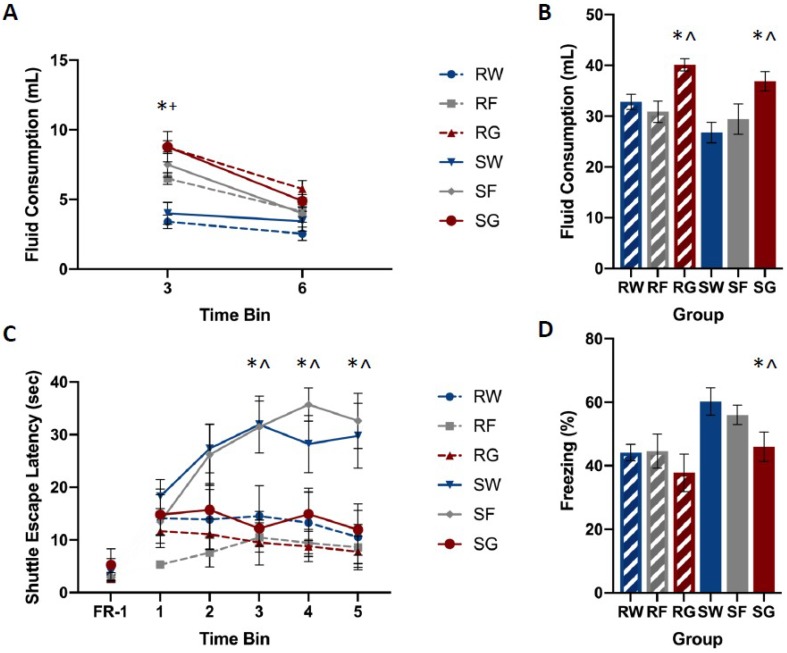
Mean fluid consumption at 3 and 6 h post-stress (panel **A**) and 18 h post-stress (panel **B**), escape latencies (panel **C**), and percent freezing for FR-1 trials (panel **D**) among groups. Rats were exposed to inescapable shock (S) or restraint (R) over a 110-min period. Rats from each stress condition had free access to water (W), a concentrated glucose solution (G), or a concentrated fructose solution (F) for 18 h, beginning immediately following stress. Shuttle-box testing occurred 24 h later. Rats were exposed to five FR-1 trials of the foot-shock. These trials were run from one side to the other shut-off shock. The amount of time spent freezing between trials was measured. Twenty-five FR-2 trials, which were broken into five groups of five, required two shuttle-crossings to shutoff shock. The time it took for required shuttle crossings was measured during each trial. Shocked animals that received glucose performed similarly to restraint controls, while animals that received water or fructose following shock exhibited increased escape latencies and freezing during testing. Rats that received glucose or fructose consumed more during the first three hours after trauma compared to their water-drinking counterparts. Rats that received post-stress glucose consumed more fluid over the 18-h period compared to rats that received water or fructose. Error bars denote mean ± SEM. * *p* < 0.05 (comparison: SG, SW), ^ *p* < 0.05 (comparison: SG, SF), + *p* < 0.05 (comparison: SF, SW).

Figure 3 shows post-stress fluid consumption, shuttle-escape latencies, and freezing among groups. Shocked groups that received water or fructose following trauma showed significantly higher escape latencies compared to the restraint controls (Figure 3A). However, the shocked group that received glucose following trauma did not show this increase in escape latency. Shocked groups that received water or fructose following trauma showed exaggerated fear responding with respect to the restraint controls (Figure 3B). However, the shocked group that received glucose following trauma did not show this increase in freezing.

A mixed-design ANOVA on FR-2 shuttle-escape latencies (Stressor × Fluid Type × Time Bin) yielded a significant interaction for Time Bin by Stressor, F(4,156) = 3.890, *p* = 0.005, and Time Bin by Fluid Type, F(8,156) = 3.914, *p* < 0.001. Newman–Keuls post-hoc comparisons (α = 0.05) indicated the following order of relationship among group means: RW = RG = RF = SG < SW = SF. A single-factor ANOVA (Stressor × Fluid Type) on FR-1 shuttle-escape latencies showed no significant main effects or interactions, F(2,50) = 1.508, *p* = 0.231.

A single-factor ANOVA on freezing (Stressor × Fluid Type) yielded the significant main effects of the Stressor, F(1,52) = 10.021, *p* = 0.003, and Fluid Type, F(2,52) = 4.612, *p* = 0.014. Newman–Keuls post-hoc comparisons (α = 0.05) indicated the following order of relationship among group means: RW = RG = RF = SG < SW = SF.

**Figure 4:**
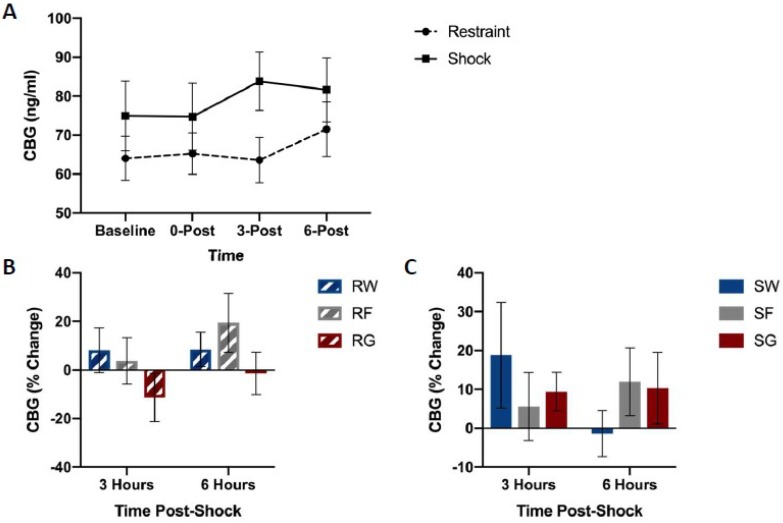
CBG concentrations between stress treatments (panel **A**), and percent change from 0 h post-stress among fluid conditions in restraint (panel **B**) or shock (panel **C**) stress treatments. Blood was collected for analysis immediately before the acute stress session, and 0, 3, and 6 h following the acute stress session. Animals received either inescapable and unpredictable shock (S) or simple restraint (R). Following the stress session, animals were given 18-h free access to a 40% glucose cocktail (G), 40% fructose cocktail (F), or water (W). CBG concentrations were not influenced by stress or fluid type.

Figure 4 shows CBG concentrations among groups. No differences were observed in CBG levels based on the fluid or the stressor type. A mixed-design ANOVA on CBG (Stressor × Fluid Type × Time Bin) yielded no significant main effects or interactions, F(2,39) = 0.309, *p* = 0.736.

**Figure 5:**
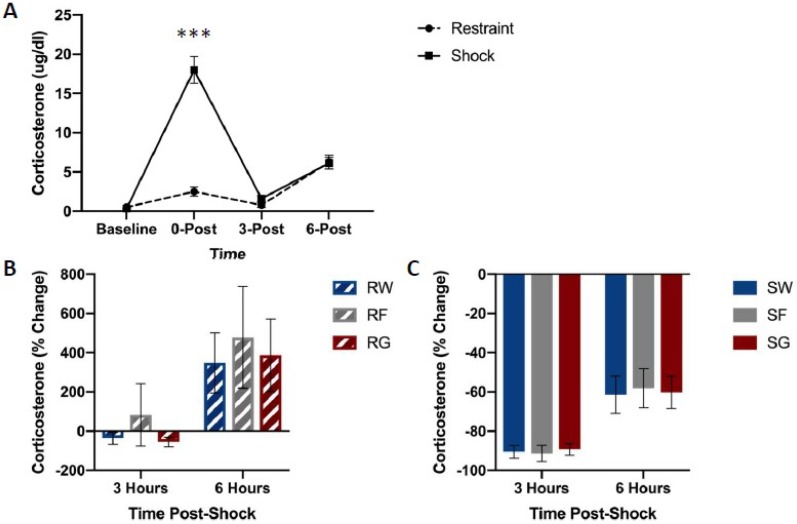
Free corticosterone concentrations between stress treatments (panel **A**), and percent change from 0 h post-stress among fluid conditions in restraint (panel **B**) or shock (panel **C**) stress treatments. Blood was collected for analysis immediately before the acute stress session, and 0, 3, and 6 h following the acute stress session. Animals received either inescapable and unpredictable shock (S) or simple restraint (R). Following the stress session, animals were given 18-h free access to a 40% glucose cocktail (G), 40% fructose cocktail (F), or water (W). Shocked animals exhibited higher concentrations of free corticosterone immediately after the stress session (0-Post). Error bars denote mean ± SEM. *** *p* < 0.001 (comparison: Restraint, Shock).

Figure 5 shows free corticosterone concentrations among groups. Shock groups showed much higher concentrations of free corticosterone compared to their restraint counterparts immediately following the termination of stress pre-treatment (Figure 5A). However, no differences were observed in free corticosterone levels based on the type of solution consumed. 

A mixed-design ANOVA on free corticosterone concentrations (Stressor × Fluid Type × Time Bin) yielded a significant Time Bin by Stressor interaction, F(1,42) = 14.618, *p* < 0.001. Post hoc analysis indicated that corticosterone concentrations 0 h after stress pre-treatment were significantly higher in rats that received shock compared to the restraint, t(47) = 7.197, *p* < 0.001.

**Figure 6:**
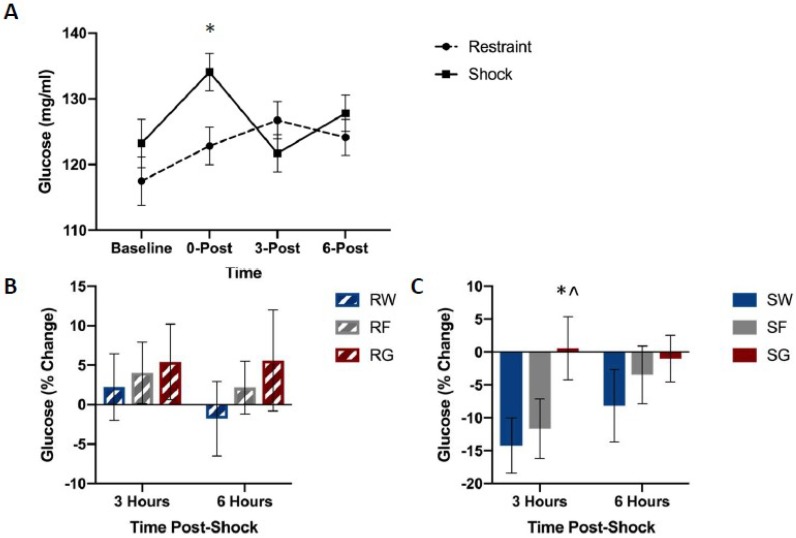
Blood glucose levels between stress treatments (panel **A**), and percent change from 0 h post-stress among solution conditions in restraint (panel **B**) or shock (panel **C**) stress treatments. Blood was collected for analysis before the acute stress session, and 0, 3, and 6 h following the acute stress session. Animals received either inescapable and unpredictable shock (S) or simple restraint (R). Following the stress session, animals were given 18-h free access to a 40% glucose cocktail (G), 40% fructose cocktail (F), or water (W). Glucose mitigated the post-stress decline in blood glucose concentrations in shocked animals. Error bars denote mean ± SEM. * *p* < 0.05 (comparison for top figure: Restraint, Shock, comparison for bottom figures: SG, SW), ^ *p* < 0.05 (comparison: SG, SF).

Figure 6 shows blood glucose concentrations among groups. Shocked groups exhibited a transient rise in blood glucose concentrations immediately following shock, followed by a dip in concentrations three hours later, which did not occur in restrained controls (Figure 6A). However, this drop did not occur in rats that received access to post-shock glucose (Figure 6C).

A mixed-design ANOVA on blood glucose concentrations (Stressor × Fluid Type × Time Bin) yielded a significant Stressor by Fluid Type by Time Bin interaction, F(2,45) = 0.894, *p* = 0.038. Post-hoc analysis indicated that glucose concentrations 3 h after stress pre-treatment were significantly higher in the SG group compared to groups SW, t(16) = 2.583, *p* = 0.020, and SF, t(17) = 2.577, *p* = 0.020.

## 4. Discussion

These experiments indicate that post-stress glucose consumption alleviates the energy homeostasis challenge of traumatic shock. It also suggests that the prophylactic effects of glucose are independent of HPA-axis activity. Furthermore, it appears that these effects are specific to glucose since fructose does not eliminate the negative behavioral consequences of stress nor does it impact blood glucose or liver glycogen concentrations in a similar way.

Figure 2 depicts corticosterone, CBG, glucose, and liver glycogen concentrations in rats that received free access to water, glucose, or fructose following traumatic stress or simple restraint. We found a large increase in both free and total corticosterone concentrations between groups that received traumatic shock compared to groups that received simple restraint. In groups that received simple restraint, there were no observed differences in free or total corticosterone concentrations between rats that received water or glucose. However, in groups that received traumatic shock, rats that received access to glucose following the acute stress session exhibited lower concentrations of free corticosterone compared to their water-drinking counterparts. No difference in total corticosterone was observed between these two groups (SW & SG). When comparing glucose to fructose in shocked rats, we observed an effect of fluid type on liver glycogen concentrations, but not corticosterone or CBG. Figure 3, Figure 4, Figure 5 and Figure 6 depict corticosterone, CBG, and blood glucose concentrations before and after stress pre-treatment. Rats in this study were also tested 24 h following stress pre-treatment for the learned helplessness phenotype. We found that glucose, but neither water nor fructose, eliminated the negative behavioral consequences of traumatic shock. Shocked rats exhibited a transient rise in blood glucose concentrations immediately following termination of the stress session, which was followed by a decline in blood glucose three hours following stress pre-treatment. This rise in glucose concentrations is most likely due to epinephrine-induced glycogenolysis [34] even though the cause of the subsequent decline is less clear. Notably, glucose, exclusively, eliminated the transient decline of blood glucose concentrations 3 h following shock. These findings show that the post-stress consumption of glucose, specifically, transiently raises blood glucose levels and mitigates liver glycogen depletion following stress exposure. Therefore, it may be this ability of glucose to reduce the metabolic challenges of stress that provide its prophylactic effects. However, how this effect works remains unclear. For example, the neural consequences of this post-stress glucose ingestion have yet to be investigated.

One potential neural pathway of glucose prophylaxis involves the hippocampus, which is a structure that is particularly vulnerable to the metabolic consequences of stress [35]. Following a stressor, CORT is one of many hormones and peptides upregulated. When, at high concentrations, CORT promotes mild insulin resistance to mobilize glucose for the brain [36]. However, not all brain regions benefit equally from this increase in circulating glucose. An increase in circulating CORT during stress impairs glucose uptake in the hippocampus and severely impairs contextual processing [12,13,37,38,39]. Furthermore, studies have shown that high CORT levels cause high levels of hippocampal atrophy compared to moderate CORT levels [40,41]. Inescapable shock in rats creates a similar neuroplastic deficit in the hippocampus [42]. Such deficits are reversed by increasing hippocampal glucose concentrations by any number of means [43]. This suggests that increasing hippocampal glucose concentrations could decrease glutamate toxicity and potentially reduce some of the sequalae of depression. This indicates that learned helplessness and PTSD-like symptoms may be in part due to the mechanism in which traumatic stress elevates cortisol levels, and that consumption of a high concentration glucose solution may moderate the CORT-dependent stress effects. 

Minor and LoLordo (1984) demonstrated that the helplessness effect is eliminated when rats can discriminate the training context, in which inescapable shock is delivered, from the shuttle-escape testing context [44]. Contextual learning critically depends on hippocampal processing [45]. Thus, post-stress glucose consumption may allow veridical encoding of the context in the hippocampus, which results in less generalization between the two contexts. This hypothesis is further supported by our previous finding that post-stress glucose only exhibits its prophylactic effects if given within the first three hours of stress pre-treatment [24]. We observed that the glucose-induced transient rise in blood glucose levels occurs three hours post-stress, which suggests that this may play a role in the beneficial effects of glucose consumption.

Perhaps the benefit of post-stress glucose is independent of hippocampal processing and is instead simply derived from its ability to prevent metabolic exhaustion. Fear is an intensely catabolic state and rapidly challenges brain metabolic homeostasis [2,8,9,10,19,46]. Under these circumstances, adenosine is released to inhibit further activity in an effort to prevent cell death. Minor and colleagues have shown that adenosine A_2A_ receptors are involved in the conservation-withdrawal symptoms normally observed following traumatic stress [2,6,8,9,10,15,19,25,46,47]. Glucose consumption following trauma might restore metabolic homeostasis, as shown by the rise in liver glycogen concentrations. This could, thereby, eliminate the necessity for the compensatory adenosine response.

The data provide evidence supporting the role of glucose in diminishing the energetic challenges of traumatic shock. These findings illustrate that consumption of glucose directly following an acute traumatic stressor reduces the transient drop of blood glucose levels following stress pre-treatment and restores glycogen in the liver. The data also suggest that the behavioral effects of post-stress glucose consumption are independent of corticosterone’s role in the induction of stress-induced behavioral effects. This indicates that glucose has a major role in mitigating the physiological and psychological challenges posed by stress, but the exact mechanism remains unclear. Lastly, it should be noted that there are several other animal models of PTSD that model many different aspects of the disease. It is possible that there are dissociable mechanisms by which different stressors induce their behavioral effects. It is, therefore, imperative to examine the behavioral and physiological effects of post-stress glucose in alternative models of PTSD in order to increase external validity and the potential for translational efficacy.
